# Physiological stress reactivity and recovery related to behavioral traits in dogs (*Canis familiaris*)

**DOI:** 10.1371/journal.pone.0222581

**Published:** 2019-09-17

**Authors:** Rian C. M. M. Lensen, Christel P. H. Moons, Claire Diederich

**Affiliations:** 1 Department of Veterinary Medicine (IVRU), University of Namur, Namur, Belgium; 2 Department of Nutrition, Genetics and Ethology, Ghent University, Merelbeke, Belgium; University of Alabama, UNITED STATES

## Abstract

This study investigated whether stress responsiveness (in one context) can be used to predict dog behavior in daily life. On two occasions (N_T1_ = 32 puppies; N_T2_ = 16 young adults), dogs’ physiological stress response after a behavioral test at home was measured in terms of reactivity (10 min post-test) and recovery (40 min post-test) for three salivary markers: cortisol, chromogranin A (CgA) and secretory immunoglobulin A (sIgA). For each marker, it was determined whether dogs with a strong physiological response displayed different behavior in daily life compared to dogs with a weaker physiological response. The results revealed three main findings: first, for CgA and cortisol, different patterns were identified according to sample time. High reactivity related to desirable traits, whereas slow recovery after the behavioral test related to undesirable traits. The findings suggest that increased levels of CgA and cortisol 10 minutes after the behavioral test reflected an adaptive stress response, whereas elevated levels 40 minutes after the test reflected unsuccessful coping. Second, patterns for sIgA differed from CgA and cortisol: significant associations were only found with behavioral traits at T2, mostly considered desirable and related to Trainability. Possibly, the delayed reaction pattern of sIgA caused this difference between markers, as sIgA reflects the (secondary) immune response to stress, due to immunosuppressive effects of cortisol. Third, predictive capacity of puppies’ physiological stress response (T1) was inconclusive, and contrary relations were found with behavioral traits at T2, suggesting that developmental factors play an important role. This study provides new insights about the relation between stress physiology and behavioral traits, and methodological advice is given to study these patterns further. In conclusion, physiological markers could provide additional insights in dogs’ tendencies to display certain behaviors, especially at the young adult stage. Further studies are needed to confirm these patterns.

## Introduction

Consistent individual-specific patterns in behavior and stress physiology have been reported in various animal species, but the relation between both types of parameters remains inconclusive (reviewed in [1,2]). It has been suggested that reactivity (i.e., an animal’s responsiveness to environmental stimuli [3,4]) is a fundamental factor shaping personality [5,6] and plays a role in various behavior problems in dogs (*Canis familiaris*) [7]. In theory, a reactive animal responds more quickly and intensely to environmental stimuli, which can be expressed by different behavioral manifestations (e.g., excitability, fear or aggression [2,7]). Accordingly, one might expect that assessment of a dog’s stress responsiveness could be used to predict its behavior in daily life. If this were true, an objective, quantifiable assessment of stress responsiveness could be a valuable indicator for the potential to develop behavior problems. An early detection of individuals “at risk” allows for timely measures to be taken to prevent behavioral problems.

To test whether stress responsiveness can be used to predict general behavior in daily life, the physiological stress response of dogs in one context should be compared to their behavioral responses in another unrelated context (e.g., [8]). However, studies on dog personality often focus on behavioral parameters (e.g., [9,10]), whereas physiological measures are more commonly used for assessing levels of stress or stress responsiveness in specific contexts (e.g., [11,12]). Furthermore, physiological parameters that are included in personality studies (i.e., assessing consistent patterns over contexts and time [13]) are generally taken on the same occasion as the behavioral parameters (e.g., [14,15]). In the current study, we assessed whether phyiological measures of stress reactivity in one context related to behavioral traits in daily life, as assessed by dog owners.

Saliva sampling is a relatively easy, non-invasive method that has often been used in dogs to monitor short-term physiological changes [16]. Cortisol is a well-known stress marker in dogs and reflects the activity of the hypothalamic-pituitary-adrenal (HPA) axis [17]. Saliva concentrations of this marker increase in response to sudden non-social stressors [18], fear-inducing events [19], and novel environments [12]. However, physiological markers are regulated through complex pathways [20,21]; and their expression could be influenced by factors like circadian fluctuations [22], physical health [23], activity [24], as well as stimulus intensity [18] and experience [25]. Hence, the measurement of multiple phsyiological parameters is recommended for a more accurate estimation of stress levels [17,26].

A more immediate response (“fight or flight”) to a perceived or actual threat is regulated through the sympatho-adrenal-medullary (SAM) axis through the release of catecholamines (adrenalin and noradrenalin). However, these markers are difficult to measure in saliva due to their low concentrations, rapid degradation, and instability in the sample [27] and are found to be poor indicators of acute sympathetic activity in humans [28]. Instead, Chromogranin A (CgA) can be measured, as it is co-released with adrenalin from the adrenal medulla and more stable in the circulatory system than catecholamines [27,29]. In dogs, plasma levels of CgA increased after physiological stress (insulin-induced hypoglycemia [29]); and increases in salivary CgA were found after psychogenic stress in humans (arithmetic task [30]), pigs (immobilization with nose snare [31]), and cows (social isolation [32]). However, little is known about this analyte as potential stress marker in dogs [33], so it was included in our study to explore its value for future studies.

Components of the immune system can also provide insights into the physiological stress response, due to the immunosuppressive effects of cortisol [23,27]. As such, markers of immune system activity provide an indirect (secondary) indication of an animal’s stress response. Decreased levels of secretory immonoglobulin A (sIgA) were found in dog saliva after acute stress [22] and 10 days after introduction into a novel kennel [34]. Therefore, this analyte was included to assess its added value to the abovementioned markers for assessing the physiological stress response.

The aim of this exploratory study was to examine if physiological indicators of stress responsiveness are related to behavioral traits in dogs as reported by the owners. For this purpose, three endocrine and immune biomarkers (cortisol, CgA, sIgA) were measured in saliva collected before and at two time points (10 and 40 min) after a behavioral test and compared to owner ratings of their dog’s behavior in daily life (by means of the validated Canine Behavioral Assessment & Research Questionnaire: C-BARQ [35]). This was done at two test stages, for the same dogs: puppy (T1) and young adult (T2). We hypothesized that a strong physiological response would be indicative of undesirable behavioral traits, based on findings that saliva levels of cortisol increased [18,19] and levels of sIgA decreased [22] after dogs were exposed to (potentially) aversive stimuli. Furthermore, we hypothesized that dogs with a longer recovery time (i.e., time before levels of physiological markers returned to baseline) would be less successful in coping with stressful situations and thus might be more prone to display undesirable (“maladaptive”) behaviors [7,36].

## Material and methods

### Subjects

Owners were recruited by participating veterinarians (N = 20) during their puppies’ first vaccination visit at the veterinary practice over a 21-month period (Table 1). The owners received two months of free dog food from a well-known brand upon completion of their participation, and the veterinarians received a gift voucher for an online general goods store.

**Table 1:** Dogs in this study and the time at which they were tested at home.

Dogs tested at T1				Dogs tested at T1 & T2			
Breed	Sex	Age	Time	Breed	Sex (neut.)	Age	Time
BSD	M	13.43	14:00	Border Collie	M (-)	16.14; 61.14	14:00
BSD*	M	22.01	14:00	Border Collie	M (-)	22.15; 60.15	10:00
X BSD / BMD	M	16.57	14:00	Border Collie	F (N)	16.72; 59.72	14:00
GSD	F	17.43	10:00	Border Collie	F (N)	18.14; 64.42	10:00
Dogo Argentino	F	18.01	14:00	X B. Collie / Husky	F (N)	13.29; 56.29	14:00
Great Dane	M	19.01	14:00	BSD	M (-)	14.72; 58.72	10:00
Rottweiler	F	15.85	14:00	BMD	F (-)	15.01; 60.00	10:00
AmStaff	F	13.57	14:00	St. Bernard	F (-)	19.58; 61:58	10:00
AmStaff	F	15.85	14:00	Dachshund	M (-)	20.99; 61.00	10:00
Engl. Cocker Sp.	M	15.43	14:00	Engl. Cocker Sp.	M (-)	14.42; 57.42	14:00
Labrador Retriever	M	21.28	10:00	Am. Cocker Sp.	F (N)	18.15; 64.29	10:00
Port. Water Dog	F	14.14	10:00	Fl. C. Retriever	F (-)	14.43; 61.43	10:00
Shih Tzu	F	21.14	14:00	Boston Terrier	M (N)	16.43; 57.43	10:00
Shih Tzu	F	14.99	14:00	French Bulldog	M (-)	18.15; 61.15	14:00
Whippet	F	19.14	10:00	X Tibetan Terrier	F (N)	21.60; 66.57	10:00
American Bulldog	F	20.14	14:00	X	M (N)	24.29; 62.29	10:00

The left panel shows dogs only tested at T1 (N = 16 puppies), the right panel shows dogs also tested at T2 (N = 16 young adults). Neuter status is only shown for T2 (puppies were not neutered). Dog age is shown for each test stage, and time of testing was the same on both occasions.

BSD: Belgian Shepherd dog, Malinois; BMD: Bernese Mountain Dog; GSD: German Shepherd dog; AmStaff: American Staffordshire Terrier; Engl. Cocker Sp.: English Cocker Spaniel; Port. Water Dog: Portuguese Water Dog; Husky: Siberian husky; Am. Cocker Sp.: American Cocker Spaniel; Fl. C. Retriever: Flat Coated Retriever; X: crossbred

(N): neutered

* No owner ratings available for this dog

At test stage 1 (T1), saliva was collected from 32 puppies (mean age ± SD: 17.57 ± 2.94 weeks) subjected to a behavioral test at home, and owner ratings were available for 31 of these puppies (procedures described below). At test stage 2 (T2), 9.91 (± 0.61) months later, 16 dogs were tested a second time at home (age: 60.85 ± 2.64 weeks), and owner ratings were available for all 16 dogs.

### Owner ratings

At both stages (T1 and T2), owners were asked to rate the behavior of their dog in daily life by means of an online questionnaire (C-BARQ: updated version of PennBARQ [35]) in Dutch or French. The C-BARQ describes 14 different behavior categories, each represented by the mean of a subset of questions (presented in [37,38]): Stranger-directed aggression (ten questions), Owner-directed aggression (eight questions), Dog-directed aggression (four questions), Stranger-directed fear (four questions), Dog directed fear (four questions), Non-social fear (six questions), Separation-related behavior (eight questions), Attachment and attention-seeking (six questions), Trainability (eight questions), Chasing (four questions), Excitability (six questions), Touch sensitivity (four questions), Energy level (two questions) and Dog rivalry (four questions). The higher the score, the more present a particular trait is in a dog according to the owner.

Owners completed the questionnaire before the test to ensure their dog’s behavior during the test would not influence their ratings. They were instructed to answer all questions but could skip a question pertaining to a situation if their dog had not experienced it yet. For any individual dog, if more than 25% of the questions for a category were missing, it was considered a missing value.

### Saliva collection & analysis

At both test stages (T1 and T2), saliva samples were collected before (pre-test), as well as 10 (post10) and 40 minutes (post40) after a behavioral test (described in S1 Appendix). In short, the first 16 minutes of this test consisted of different social situations (e.g., entrance of a stranger, being ignored by persons in the room, being left alone), and in the second part (27 min), the dogs were exposed in a random order to several stimuli with varying visual and acoustic properties (e.g., sound of a barking dog, squeaky toy, weasel ball, vacuum cleaner). Two startling stimuli were presented at the end (umbrella opened suddenly and shaking of a metal cylinder containing keys), so that the overall test remained as little stressful as possible to the dogs.

The test was conducted in a familiar room at home to minimize influences of unfamiliar settings on the stress response [19,39]. To minimize influences from circadian fluctuations, testing only took place at one of two predetermined time points (10:00 or 14:00), and, on both occasions (T1 & T2), the dogs were tested on the same day (i.e., with a similar routine).

Saliva samples were collected according to a standardized protocol that was refined over several pilot trials [16]. Owners habituated their dogs to the sample procedure, starting four days before the test (S1 Appendix), and collected the saliva samples to prevent influences of unfamiliar persons [19,40]. On the test day, owners withheld food from the dogs at least one hour before sampling and did not let the animal drink or engage in strenuous activities at least half an hour beforehand to prevent contamination of saliva samples and influence of physical activity on the physiological measures [41,42]. At each sampling occasion (pre-test, post10, post40), the owners put on clean latex gloves and collected saliva samples with four swabs. They put one swab in each cheek pouch and gently held the dog’s muzzle for one minute, after which they placed the two swabs back in their centrifugation tubes. Immediately after, they took samples with the two remaining swabs. The owners were instructed not to pet the dog during sampling, as this could influence the physiological response [43]. The dogs were not restrained, and sampling was stopped if an animal appeared overly stressed by the procedure (e.g., low posture, avoidance, resistance). Sampling occurred within four minutes to prevent handling from influencing marker concentrations [44]. Owners noted sampling time, sampling duration, last eating/drinking time, activity before sampling, and any additional remarks. They then placed the four centrifugation tubes, containing one swab each, in the freezer until the end of the behavioral test.

After the test, the samples were transported on ice to the tester’s home and stored within two hours (-20°C). Within three months [42], samples were transported on ice to the University of Namur, where they were centrifuged at 3000 rpm (1851.41 x g for 15 min at 10°C) and samples containing blood or other visible contamination were discarded (N = 61 or 11.30%; from an additional 32 samples [5.92%] no saliva could be extracted). The extracted saliva volume per swab was noted, then saliva was pooled per sampling occasion (four swabs), resulting in three samples per test day: pre-test, post10, post40. The samples were assayed for the following three analytes, given sufficient saliva was available (S1 Table): CgA, cortisol, sIgA (respective kits: Human Chromogranin A EIA kit, Yanaihara Institute, Shizuoka, Japan; Salivary cortisol enzyme immunoassay kit, Salimetrics LLC, State College, Pennsylvania; Dog IgA ELISA Quantitation set, Bethyl Laboratories, Montgomery, Texas). Samples had to be diluted to assay CgA (1:8) and sIgA (1:3000), so the required sample volumes for duplicate measurements were 8 μl, 50 μl and 3 μl for CgA, cortisol and sIgA, respectively [16].

To minimize assay influences, all samples from a dog (collected on one test day) were assayed on the same ELISA plate, and at both test stages the same dogs were assayed together on a plate. For example, if at T1 the ELISA plate contained samples from dogs 1–4 then the ELISA plate at T2 also contained samples from dogs 1–4. Intra-assay coefficients of variation (CV) were calculated by dividing the standard deviation of duplicate measurements of a sample by the mean of these measurements. Samples with an intra-assay CV > 15% were excluded from further analysis (N = 5, 0 and 16 for CgA, cortisol, and sIgA, respectively), so that analytical imprecision was less than half of the within-subject variation [45]. Mean intra-assay CVs for the final samples were 4.56% (CgA), 5.16% (cortisol) and 4.58% (sIgA). Inter-assay CVs, based on repeated measurements of two control samples (aliquoted before freezing) were relatively high: 67.90% (CgA), 11.60% (cortisol), and 41.06% (sIgA). However, over the whole assay period (almost two years), the control samples were frozen longer than the recommended 6 months [42], so instability of the analytes might have contributed to this high variance. Acceptable inter-assay CVs of 9.31% (CgA), 4.98% (cortisol), and 6.38% (sIgA) were found for the same assay kits during pilot trials [16]. Each dog served as its own control, and all samples from that dog (one test day) were assayed on the same plate, so inter-assay variation is expected to minimally influence the measured values for each dog (expressed as *changes* in value over time, see “statistics”).

### Ethics considerations

The described procedures (behavioral test, saliva sampling) were approved by the Ethical Commission of the University of Namur, which did not consider them animal experiments based on the non-invasive nature (as attested by seven international researchers, experienced with these types of measurement). The protocol was agreed upon by the Belgian State authority for Directive 2010/63/EU.

The owners were informed about the study aims and procedures and signed a letter of informed consent. Their participation was voluntary, and they were told that they could interrupt the behavioral test at any moment. Also, endpoints were formulated for the behavioral test (see S1 Appendix) to protect the welfare of the dogs.

### Statistics

As high inter-individual variation in marker concentrations may confound interpretation of reactivity and recovery parameters [46], we analyzed differences between time points, rather than absolute concentration values. Pre-test values were subtracted from post10 and post40 values [47] to create two variables: Δ10, Δ40.

Given the low sample size (see S1 Table), and in consultation with a statistician, non-parametric analyses were used to identify potential patterns between physiological and behavioral parameters. Per marker and for each variable, the dogs were median-split to distinguish those with the largest physiological response from those with the smallest response. Individuals were considered to have a “strong response” if their saliva concentrations *increased* above median for cortisol and CgA or *decreased* below median for sIgA [22]. Scatter plots were used to verify if the median split the sample into two distinctive groups. For three variables, a dog was moved from the “weak response” to the “strong response” group (T1: CgA Δ40; sIgA Δ10) or conversely (T2: CgA Δ10) because their values resembled more closely to the newly assigned group. The first author then used a Mann-Whitney U Test (IBM^®^ SPSS^®^ Statistics, Version 20) to determine whether these groups differed significantly (α < 0.050) for the different C-BARQ scores at T1, T2 and when comparing physiological responses at T1 with behavioral scores at T2.

## Results

The following sections describe significant associations. To minimize reporting bias [48], all tested associations and ranges in marker concentrations are reported in a supplemental table (S2 Table).

### Associations between physiological and behavioral variables at the same test stage

For CgA and cortisol, significant associations were found with behavioral traits at both test stages (Table 2). Puppies with a strong physiological response 10 minutes after the behavioral test (Δ10) received lower scores for Separation-related behavior (CgA), whereas those with a slow recovery (Δ40) received lower scores for Trainability (CgA) and higher for Dog Rivalry and Stranger-directed fear (cortisol). These results suggest that dogs with a strong physiological response shortly after the test were less likely to display undesirable behaviors upon separation. This finding is in contrast with our hypothesis, as we expected *more* undesirable behaviors for dogs with a strong physiological response (both for Δ10 and Δ40). Indeed, we found that a strong physiological response *40 minutes* after the test was associated with *more* undesirable behaviors in daily life, as reported by the dog owners.

**Table 2:** Significant differences in C-BARQ scores (owner ratings) for dogs with small/large physiological changes in response to the behavioral test (Mann-Whitney U test).

Marker	Physiol. change	C-BARQ	Weak response (median ± IQR)	Strong response (median ± IQR)	U	P	N	ES
CgA	Δ10 _T1_	SEP_T1_	1.07 ± 0.39	0.63 ± 0.25	17.5	0.02	19	0.83
	Δ40 _T1_	TRA_T1_	2.57 ± 0.82	2.38 ± 0.25	18.0	0.04	18	0.78
Cortisol	Δ40 _T1_	RIV_T1_	0.00 ± 0.00	1.00 ± 1.00	3.5	0.04	10	0.86
	Δ40 _T1_	SDF_T1_	0.00 ± 0.00	0.25 ± 0.25	14.5	0.03	17	0.80
CgA	Δ10_T2_	SDF_T2_	0.75 ± 1.00	0.00 ± 0.00	2.0	0.02	10	0.76
	Δ40 _T2_	EXC_T2_	2.58 ± 0.92	1.42 ± 0.59	0.0	0.02	8	1.00
Cortisol	Δ10 _T2_	TRA_T2_	2.38 ± 0.63	3.13 ± 0.44	0.5	0.03	8	0.97
	Δ40 _T2_	NRG_T2_	1.50 ± 1.75	3.00 ± 0.50	0.0	0.02	8	1.00
sIgA	Δ10 _T2_	TRA_T2_	2.63 ± 0.38	3.13 ± 0.38	0.0	0.03	7	1.00
	Δ40 _T2_	TRA_T2_	2.50 ± 1.00	3.13 ± 0.38	0.0	0.05	6	1.00
	Δ40 _T2_	NSF_T2_	1.50 ± 0.50	0.67 ± 0.67	0.0	0.05	6	1.00
	Δ40 _T2_	TCH_T2_	1.50 ± 0.75	0.25 ± 0.75	0.0	0.05	6	1.00

The upper panel presents data from T1 (puppy stage), the lower panel from T2 (young adult stage). “Strong response” dogs are those with the largest physiological change (above median), as expected for that marker (increase for CgA and cortisol, decrease for sIgA).

Δ10 / Δ40: change in salivary marker concentration 10/40 min after the behavioral test, compared to pre-test

SEP: Separation-related behavior; TRA: Trainability; RIV: Dog rivalry; SDF: Stranger-directed fear; EXC: Excitability; NRG: Energy level; NSF: Non-social fear; TCH: Touch sensitivity

ES: effect size (probabilistic index)

At T2, similar patterns emerged for CgA and cortisol. High reactivity (Δ10) was associated with desirable behaviors in daily life: low Stranger-directed fear (CgA) and high Trainability (cortisol). In contrast, a slow recovery (Δ40) was associated with low Excitability (CgA) and high Energy level (cortisol), which might (arguably) be considered undesirable by owners (see discussion).

In contrast to the primary stress markers cortisol and CgA, significant associations between sIgA levels and behavior were only found at the young adult stage (T2), and only for desirable traits. A strong physiological change in sIgA levels, both at 10 and 40 minutes after the test, was associated with the desirable trait Trainability. In addition, slow recovery after the test (Δ40) was associated with low levels of Non-social fear and Touch sensitivity.

### Associations between physiological variables at T1 and behavior at T2

Ambiguous associations were found between puppies’ stress response, measured by levels of cortisol and CgA at T1, and their future behavioral traits (T2). Puppies with high physiological reactivity (Δ10) at T1 received higher scores at T2 for Stranger-directed fear, and Excitability (CgA) and lower scores for Attachment and attention-seeking (cortisol; Table 3). Those puppies with sustaining levels of CgA after the test (Δ40) received higher scores for Excitability and Energy level later in their life.

**Table 3:** Significant differences in C-BARQ scores (owner ratings at T2) for puppies with small/large physiological changes in response to the behavioral test (Mann-Whitney U test).

Biomarker	Physiol. change	C-BARQ	Weak response (median ± IQR)	Strong response (median ± IQR)	U	P	N	ES
CgA	Δ10 _T1_	SDF_T2_	0.00 ± 0.50	1.75 ± 2.25	4.0	0.01	13	0.91
	Δ10 _T1_	EXC_T2_	2.17 ± 1.66	3.25 ± 1.33	4.0	0.02	13	0.91
	Δ40 _T1_	EXC_T2_	1.67 ± 0.50	2.83 ± 1.16	5.0	0.04	12	0.71
	Δ40 _T1_	NRG_T2_	2.00 ± 0.50	3.00 ± 1.00	4.5	0.03	12	0.71
Cortisol	Δ10 _T1_	ATT_T2_	2.67 ± 1.00	2.17 ± 0.17	5.0	0.04	12	0.85
sIgA	Δ10 _T1_	TRA_T2_	2.38 ± 1.00	3.07 ± 0.50	3.0	0.03	11	0.83
	Δ40 _T1_	DDF_T2_	0.00 ± 0.25	1.25 ± 0.50	0.0	0.02	8	1.00

“Strong response” dogs are those with the largest physiological change (above median) as expected for that marker (increase for CgA and cortisol, decrease for sIgA).

Δ10 / Δ40: change in salivary marker concentration 10/40 min after the behavioral test, compared to pre-test

SDF: Stranger-directed fear; EXC: Excitability; NRG: Energy level; ATT: Attachment and attention-seeking; TRA: Trainability; DDF: Dog-directed fear

For sIgA, again a positive association was found between large physiological reactivity following the test (Δ10) and the desirable trait Trainability. In contrast, a slow recovery (Δ40) at the puppy stage (T1) was associated with more Dog-directed fear later in life (T2).

## Discussion

This study investigated whether stress responsiveness, as measured via physiological markers in one context were related to dogs’ general behavior in daily life (as rated by their owners). At two stages (T1: puppy, T2: young adult), the dogs’ physiological stress response after a behavioral test at home was measured in terms of reactivity (10 min post-test) and recovery (40 min post-test) for three salivary markers: cortisol, chromogranin A (CgA) and secretory immunoglobulin A (sIgA). Based on these values, it was determined whether dogs with a strong physiological response (for each marker separately) displayed different behavior in daily life, compared to dogs with a weaker physiological response. We hypothesized that a strong physiological response 10 minutes after the test would be associated with undesirable behavioral traits, and we expected the same for a longer recovery time (i.e., time before physiological levels return to baseline). The results revealed three main findings: 1) for CgA and cortisol, different patterns were identified according to sample time post-test, 2) patterns for sIgA differed from CgA and cortisol, and 3) predictive capacity of puppies’ physiological stress response (T1) was inconclusive, and ambiguous relations were found with behavioral traits at T2.

First, for CgA and cortisol, high reactivity at 10 minutes after the behavioral test related to desirable traits, whereas slow recovery 40 minutes after the test related to undesirable traits. The former result suggests that dogs with a relatively strong physiological response following the test respond to stressors in an adaptive manner [23,49], reflected in daily life by less fear and higher trainability. In contrast, the latter result might indicate unsuccessful coping (slow recovery), as reflected by the associations with undesirable traits. This could also explain the high Energy (cortisol) and low Excitability (CgA) scores at T2. Increased activity can be a mechanism to cope with prolonged stress [24,50], and, if the dogs were in a negative emotional state, they may have responded less excitedly to the situations described in the C-BARQ score Excitability (e.g., going for walks, arrival of visitors, owner coming home).

The finding that associations of physiological responses with behavioral traits vary according to the sample times indicates the importance of measuring *changes* in marker concentrations, at *multiple* time points. Collection of baseline samples before the onset of a specific event allows for a correction of inter-individual variation in marker concentrations [46] and thus provides a more accurate indication of each dog’s physiological response to that event. This is supported by findings from Wormald et al. [8], who only found a significant difference between dogs that either passed or failed an aggression test, when considering the *change* in salivary cortisol levels (pre-post venipuncture). Likewise, Sherman et al. [15] found only significant associations between “emotional reactivity” (measured in a behavioral test) and cortisol levels in saliva and plasma when considering the *change* compared to baseline but not when using absolute post-test values.

Furthermore, the collection of multiple samples is necessary to monitor changes over time, thereby distinguishing dogs with a high (initial) reactivity from dogs with a more blunted (prolonged) stress response. In fact, we hypothesized that a strong physiological response (in itself) would be indicative of undesirable behavioral traits [2,19]. Instead, our findings suggest that the recovery time tells us more about a dog’s overall stress responsiveness. Possibly, dogs that are more prone to stress in daily life have frequent elevations of stress markers, which might have led to an attenuated stress response due to negative feedback [21]. In line with this, Beerda et al. [51] found that dogs (N = 8) subjected to 5 weeks of social and spatial restriction, showed an increase over time in daily cortisol levels but had an attenuated cortisol response when exposed to a noise stressor. Dreschel and Granger [19] found that dogs living with other dogs had a lower increase in cortisol levels 20 and 40 minutes after exposure to a fearful event (thunderstorm recording), and their baseline cortisol levels tended to be higher. They argued these findings might reflect stress in daily life from living with another dog. Likewise, Rosado et al. [52] found higher plasma levels of cortisol in dogs diagnosed as aggressive, which they attributed to stress most likely resulting from an inconsistent and unpredictable living environment. Translating these findings to our study, it is possible that relatively small changes in marker concentration shortly after the behavioral test reflected an attenuated stress response to that specific event.

Second, this study revealed differences among physiological markers. Contrary to findings for cortisol and CgA, changes in sIgA levels only related to behavioral traits at T2, most of which were considered desirable. In particular, Trainability was positively associated with reactivity and recovery at T2 and also predicted by high reactivity at T1. Likewise, Kikkawa et al. [53] found sIgA levels to differ significantly between dogs (11–14 months old) selected (N = 25) or rejected (N = 44) for guide dog training. During a 2-week assessment period, the dogs were kenneled and their sIgA levels were measured on days 1, 2, 3, 7 and 14. The sIgA levels of subsequently selected guide dogs gradually increased, whereas sIgA levels of rejected dogs remained relatively low, despite similar housing and caring conditions. On day 14 the selected dogs had significantly higher sIgA levels than rejected dogs, which the authors interpreted as a difference in “adaptive ability”. These findings seem to reveal the same pattern as described above for cortisol and CgA (Δ40): consistently low sIgA levels suggest that the rejected dogs had low adaptive capacity, with slow recovery during a period of prolonged stress (kenneling). In our study, however, a strong response (i.e., relatively low levels of sIgA) was associated with *high* Trainability scores, which appears to be more in line with the Δ10 results for CgA and cortisol. These contrary findings are likely a result of different protocols: in our study the samples were collected after startling stimuli (two at the end of the test), whereas Kikkawa et al. [53] took samples at predetermined time points, not associated with a particular stimulus. Consequently, sIgA measures in our study appear to reflect the more immediate response to a stressor, in contrast to the long-term measures of Kikkawa et al. Indeed, in an extensive review on stress and secretory immunity in humans, Bosch et al. [54] identified an overall distinction between acute and chronic stress: chronic stress generally induces a *decrease* in sIgA levels, acute stressors mostly induce an *increase*. One would then expect the dogs from our “strong response” group to be more prone to chronic stress, and thus have a *lower* trainability (as found by Kikkawa et al. [53]). On the other hand, if we take into account a possible attenuation in cortisol response for dogs under chronic stress (as described above), it is possible that this results in less immunosuppression, hence relatively *higher* sIgA levels, in these dogs after an acute stressor. If this were true, then this would explain why the “weak response” group had relatively low Trainability scores. However, many factors affect the findings (including type of stressor, timing of sampling [22], and the balance of sympathetic-parasympathetic control [27,54]. Consequently these complex interactions need to be further explored and elucidated. Nevertheless, it is noteworthy that we found three associations with the Trainability trait that were consistent over time, and these findings along with those from Kikkawa et al. [53] suggest that sIgA holds potential as a marker of (long-term) coping ability. Further investigation is needed to unravel individual differences in sIgA levels, both in response to short-term and long-term stressors.

The differences between CgA and cortisol on one hand and sIgA on the other hand are in line with findings from other studies, and distinguishes them as primary and secondary markers of stress. Similar reaction patterns were found for CgA and cortisol in dog plasma [29], pig saliva [31] and human saliva [55]. Svobodová et al. [56] reported no significant association between salivary cortisol and sIgA values after a stressful condition, though Skandakumar et al. [34] reported a negative (semi-logarithmic) relation between cortisol and sIgA levels in repeated saliva samples from six dogs. Possibly these contradictory findings reflect the delayed reaction pattern for sIgA, in comparison to cortisol: in response to a stressor cortisol is first released, which subsequently inhibits components of the immune system [27], thereby decreasing sIgA levels. So, strictly speaking sIgA is a marker of immune response, which responds to stress, instead of a primary stress marker. The delayed reaction pattern for sIgA could explain the correlations with desirable behavioral traits and sIgA measured 40 minutes after the behavioral test (as opposed to cortisol and CgA). After a stressor sIgA levels return to baseline only after 30–60 min [22]. Consequently, 40 minutes after the test might have been too soon to detect differences between dogs in coping capacity (recovery), as we found for CgA and cortisol. Unfortunately, combined analyses with all markers (e.g., pairwise comparisons; Principal Component Analysis) were not possible due to incomplete datasets for the individual markers (S1 Table). So, more research is needed to further elucidate the interaction between different stress markers.

Third, predictive capacity of stress physiology at the puppy stage (T1) remains inconclusive. Apart from Trainability, sIgA responsiveness in puppyhood only related to an *undesirable* behavioral trait at T2 (Dog-Directed fear), in contrast with the findings described above. Only one association was found for cortisol, and contradictory associations were found for CgA: Stranger-directed fear at T2 was associated with *high* reactivity (Δ10) at T1 but with *low* reactivity at T2. Likewise, Excitability at T2 was associated with *slow* recovery (Δ40) at T1 but with *fast* recovery at T2. These contradictions could partly be explained by the different datasets at both test stages, as the findings describe *relative* patterns for each test population [57]. For instance, a dog’s stress response may have been relatively strong compared to the other dogs at T1, but the same response may have been relatively weak at T2. Nevertheless, it is interesting that associations with the same behavioral traits were found at both test stages, even though inverted. Probably the relation with behavioral traits is shaped by developmental factors [43,58], and further studies should elucidate the predictive capacity of the described salivary markers.

### Limitations

One important limitation of this study is the small sample size. Data were inevitably lost during laboratory analyses (insufficient or contaminated saliva samples) and by excluding samples with an intra-assay CV > 15%. Effect sizes were relatively high, but this is partially caused by the small sample size (i.e., the denominator of the probabilistic index). Furthermore, potential influences like breed, sex, or neuter status [40,59] could not be analyzed, so the results should be interpreted with caution. Though the patterns identified in this study should be confirmed, they highlight important considerations for future studies.

Another limitation of this study is the high dependence on dog owners. Participation rate was low for this longitudinal and multidisciplinary study, despite proactive recruitment by veterinarians and offering incentives. Furthermore, not all owners adhered equally well to the specific test and sampling procedures. This may have caused inter-individual variation and possibly also some intra-individual variation. By median-splitting the group, the influence of such variation was expected to be minor: individuals with a large stress response would fall into the “strong response” group regardless of the exact change in marker concentration and vice versa. However, the use of the median was an arbitrary choice, and the created groups might not reflect ‘true’ differences in stress response. Therefore, the data were visually explored to ensure they were split into two distinctive groups.

Furthermore, dogs that would normally cope by means of owner interaction, activity or play had limited options for recovery, as interaction and activity were limited for standardization. Nevertheless, the saliva samples still provided valuable information about their coping ability when owner support or outlet options are limited. For interpretation of the results, standardization of the test situation was thus preferred. For future studies, it might be useful to note signs of stress at the time of sampling, instead of merely activity, and to question owners how their dog usually copes in a stressful situation. This would allow retrospective inspection whether or not the dogs recovered, and if not, whether this might have been caused by the absence of relevant maintenance stimuli [60]. Alternatively, the potential value of hair samples to determine dogs’ general stress responsiveness could be further explored. Although hairs contain a mixture of analytes accumulated over weeks, it might give an estimation of dogs’ stress reactivity, when taking into account activity levels and coat color [61,62].

Another potential influence concerns the baseline levels before the test. The owners met the tester outside 20–30 min before the test but the dogs’ reactions to their owners’ return could not be controlled. Excited dogs might have had higher baseline levels of the measured markers, which would hypothetically result in lower differences between post-test and pre-test marker concentrations. However, no such patterns were identified in the dataset, and the findings described above appear to reflect plausible differences in reactivity. This suggests that the influence of different baseline levels was minor.

## Conclusions

The findings of this exploratory study suggest that individual canine differences in physiological stress response relate to behavioral traits, mainly describing fearfulness, reactivity or responsiveness to training. Different patterns were found for physiological stress reactivity and recovery, suggesting that both are important for determining dog stress response and coping ability. High reactivity in response to a stressor is ambiguous to interpret, as it could indicate an adaptive or a maladaptive response. Slow recovery, on the other hand, reflects a maladaptive response. Differences in markers were also found, confirming that a combined analysis of multiple endocrine and immune biomarkers would give a more accurate indication of stress levels [17,26]. Finally, predictive capacity of puppies’ physiological stress response was inconclusive, and more research is needed to assess the influence of developmental factors. Though these tenuous conclusions are based on a relatively small dataset, they shed new light on the interpretation of physiological markers and their application in future studies. Hopefully, our findings will inspire other researchers to measure both reactivity and recovery and to use multiple markers for a more accurate indication of the physiological stress response. Further research is needed to confirm these patterns in a larger population.

## Supporting information

S1 Appendix Description test procedures. (PDF)

S1 Data Raw data used for statistical analyses, and legend on second tab. (XLSX)

S1 Table Overview of collected saliva volume and measured variables. (PDF)

S2 Table Tested associations (Mann-Whitney U test) between dogs’ physiological responses and C-BARQ scores. (PDF)
