# Supplementary material for: Physiological stress reactivity and recovery related to behavioral traits in dogs (Canis familiaris)
Source: PLoS One. 2019 Sep 17;14(9):e0222581. doi: 10.1371/journal.pone.0222581 (PMC6748563; doi:10.1371/journal.pone.0222581)
Supplement: S1 Appendix — (PDF) [file pone.0222581.s001.pdf]

Appendix: description test procedures

Rian C.M.M. Lensen, Christel P.H. Moons, Claire Diederich

1. Preparations before the test day

The dogs were tested in a familiar room, to minimize influence of unfamiliar settings on the stress response (Dreschel & Granger 2005; van den Berg et al., 2006). The room had to have a non-transparent door, so that the tester could leave in between subtests. The characteristics of the room (e.g., size, furniture, windows) varied between houses, but the owners received instructions to standardize the setting as much as possible (Fig. S1) by organizing the room several days before the test (step by step, preventing sudden changes in the dog's environment).

Owners received material by mail to habituate their dogs and themselves to the procedure of saliva sampling, starting four days before the test (Fig. S2). For the first three days, the owners were asked to habituate their dogs with cotton buds (150 x 4 mm, WA 2 PL; Heinz Herenz Medizinalbedarf GmbH), as demonstrated in an instruction video. These devices were chosen, as the handle made them particularly easy to use, even for unexperienced persons (Lensen et al., 2015). Four days before the test day, owners were asked to only shortly insert one cotton bud, provided the dog accepted it. Then, they could gradually increase the duration the cotton bud was in the dog's mouth until about one minute. On the subsequent days the owners used two cotton buds (one in each cheek). The owners were also asked to put a dummy Polar® chest strap around the dog's chest, increasing the duration 10 minutes each day. However, cardiac data were deemed unsuitable for further interpretation during the data processing stage, and will not be further discussed in this appendix, nor in the research article it relates to.

One day before the test day, the owners imitated the procedure that would be used on the actual test day. They practiced saliva sampling with latex gloves and synthetic swabs (Salimetrics Children's Swab; Salimetrics Europe Ltd.; cut in three) that were used during the actual sample collection. They were encouraged to give their dogs a treat after these preparatory steps, to create a positive association with the devices used and to stimulate saliva flow during subsequent saliva sampling (Dreschel and Granger, 2005). They were instructed to present the treat in a closed fist, and only feed it to the dog once it touched the fist, as preparation for the subtest 'FIST' (described in Table S1).

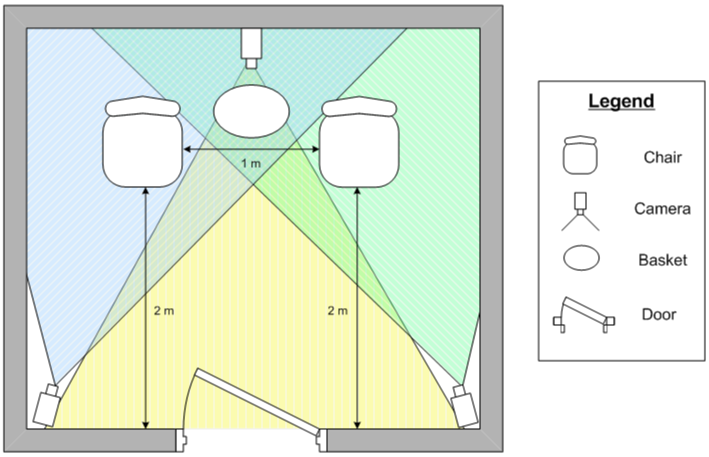

Fig. S1 Schematic overview of the test room set-up. The room was familiar to the dog and gradually prepared by the owner from a few days before the test.

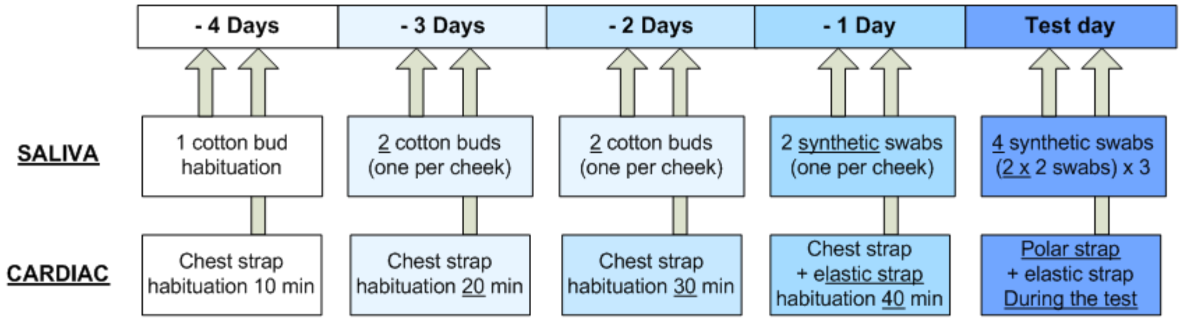

Fig. S2 **Preparatory steps the owners had to perform to habituate their dogs and themselves to the procedures on the test day. Different shades and underlined text indicate a change in procedure compared to the preceding day.**

2. On the test day

2.1. Preparations before the test

On the test day, owners were instructed to withhold food from the dogs at least one hour before saliva sampling, and to not let the animal drink or engage in strenuous activities at least half an hour beforehand, to prevent contamination of saliva samples and influence of physical activity on the physiological measures (Glenk et al., 2014; Salimetrics LLC and SalivaBio, 2013). Upon arrival, the tester (R. Lensen) phoned the owners to signal that they could come outside to collect the test material. She stayed outside to remain unfamiliar to the dog (‘stranger’), while the owners prepared for the test (within 30 minutes), as described in a small instruction booklet. The owners first put on latex gloves and took pre-test saliva samples from their dogs. They put one swab in each cheek pouch and gently held the dog’s muzzle for one minute, after which they placed the two swabs back in their centrifugation tubes. Immediately after, they took samples with the two remaining swabs. Owners were instructed not to pet the dog during sampling, as this could influence the physiological response (Hennessy et al., 1998). The habituation phase and the treats given to the dogs during that phase (but not on the test day), were intended to reduce handling stress for the dogs. The dogs were not restrained, and sampling was stopped if an animal appeared overly stressed by the procedure (e.g., low posture, avoidance, resistance). Sampling occurred within 4 min to prevent that handling itself influenced the concentration of stress markers under study (Kobelt et al., 2003). Owners noted the time of day (start of sampling), the duration the swabs had been in the mouth, the duration the dog had not eaten or drunk, whether there was activity before sampling and any additional remarks. After putting the samples in the freezer, the owners placed a Polar® chest strap on the dog for cardiac measures (Polar® RS800CX with Wearlink), and wrapped an elastic strap around it to keep it in place (Petflex®, 7.5 cm x 4.5 m, red; Andover Healthcare, Inc.). They then installed cameras to film the behavioral test (Sony®: DCR-SR50E, HDR-CX570, HDR-CX200E), and informed the tester by phone when all preparations had been done. The tester waited for another 5 minutes (neutral situation for the dog) before ringing the doorbell (start of the behavioral test, within 30 min after saliva sampling).

2.2. Behavioral test

The first part of the test (Table S1, Part A; total duration = 16 min) measured dogs' responses to different social situations, and the second part (Table S1, Part B; total duration = 27 min) measured dogs' responses to several stimuli with varying visual and acoustic properties. These stimuli were presented in a random order, but the two startling stimuli were always presented at the end (first, an umbrella was suddenly opened, then a metal cylinder containing keys was shaken), so that the overall test remained as little stressful as possible to the dogs. Each subtest, except for manipulation of the paws, lasted for one minute (following King et al 2003), after which the tester left the room along with the presented stimulus. A two-minute pause followed each subtest to minimize carry-over effects of the preceding stimulus (De Meester et al., 2011) and to allow the dog to recover if necessary. As owner presence can influence a dog's behavior (De Meester et al., 2011; Palmer and Custance, 2008), the owner remained in the room throughout the test, but was only allowed to interact with the dog during specified subtests (Table S1: entering owner, manipulation, treat in fist). They were instructed to remain seated, without looking explicitly at the dog, and not to talk or attract the dogs' attention towards the presented stimulus. In the period between the subtests the owners could only interact with their dog if the dog actively sought attention (ignoring attention-seeking behavior might lead to frustration and prevent recovery from the previous subtest). To standardize the owners' behavior they were encouraged to read the small instruction booklet during the test, providing in depth information about the test procedure.

To measure dogs' spontaneous reactions to the stimuli, they could move freely in the test room, except during the last two startling subtests, when the dogs were put on a leash. The owners put on their dogs' usual leash and were instructed to keep it loose, so that the dog could move freely. The leash was a precaution in case the dogs would react strongly (e.g., snap) during presentation of the startling stimulus. If the dog moved away for the complete length of the leash, the owner was instructed to let go. As an extra precaution, the tester wore protective clothing (sleeve, leg), underneath oversized normal clothing, during the whole test. All test objects, as well as the tester's shoes, were thoroughly cleaned after each test with a non-toxic cleaning product (Hygiënische Multi-Reiniger, Dettol®, Hoofddorp, The Netherlands) to remove scents from tested dogs. Tester's cloths were always washed with the same product (Robijn Wol en fijn, Unilever Nederland B.V., Rotterdam, The Netherlands) and sprayed with clothing freshener (Robijn Refresh, Lever Fabergé, Bodegraven, The Netherlands) to ensure a similar odor each time.

For a reliable measurement of the dogs' behavioral traits, the same test protocol was used at both test stages (Fratkin et al., 2013). As this inevitably changed the novelty aspect of the presented stimuli (Svartberg et al., 2005; van der Borg et al., 2010), these stimuli were slightly adapted on the second test occasion (Table S1). The same was true for the tester, who wore a cap and a pony tail, glasses and slightly different clothing to appear unfamiliar upon entrance. She also wore different perfumes: Guess Gold at the puppy stage and Jil Sander Sport for women at the young adult stage. The dogs were tested at T2 on the same day (i.e., with a similar routine) and the same time (10:00 or 14:00), as they had been tested at T1.

Table S1 Description of the behavioral subtests and their duration (s), specifying the different stimuli used at the puppy (T1) and young adult (T2) stage.

Subtest	(s)	Subtest description
Part A: social subtests (fixed order; no pauses in between)		
DOOR	60	T rang the doorbell (/knocked three times on the door), O opened the door as usual (greeting T), then both persons remained silent in the corridor (dog remained in the test room)
STR	30	T entered the room and after 10 s greeted the dog for 20s: 'hello [name of dog]', petting the head and back (if the dog accepted it) / called the dog's name while tapping with both hands on her legs (repeated every 5 s until dog came)
IGNS	270	T sat down and ignored the dog (watching the timer)
OWN	30	O entered the room and after 10 seconds greeted the dog, as described above
IGNB	270	O sat down next to T and both persons ignored the dog (O read the instruction booklet)
ALON	300	Both persons left the house (like O would usually do, e.g., taking keys, putting on coat, saying goodbye to dog), dog remained alone in the test room (start of subtest = closing of the door of the house)
Part B: various stimuli (randomized, except for last two); interval between stimuli: 3 min		
DOG	60	T (in the corridor) played for 30 s the sound of a dog barking at a stranger at his home (Maros et al., 2008; Pongrácz et al., 2005) with the corridor door a chink open (left open after previous subtest). After 30 seconds of silence, T closed the door again<br>*T1/T2: similar (but different) recordings*
SQUE	60	T squeezed the toy and threw it in the dog´s direction<br>*T1: rhinoceros / T2: elephant (both grey, equal sizes: 10 cm)*
BALL	60	T called the dog and rolled a ball in its direction<br>*T1: red rubber ball (ø 6 cm) / T2: blue & yellow tennis ball (ø 7 cm)*
WEAS	60	T activated an automatically rolling weasel ball and put it on the floor, after 30 s T deactivated it an left it on the floor (30 s)<br>*T1: original plush toy (brown, with black stripes) / T2: black toy of similar length (25 cm)*
CAR	60	T made a remote-controlled car repeatedly approach the dog for 30s, after which it remained immobile (30 s)<br>*T1: pick-up Silverado / T2: jeep Wrangler (both: red, length:20 cm; New Bright)*
VAC	60	O vacuumed for 30 s, then turned the machine off and sat down next to T (remaining passive for 30 s)<br>*T1: green, no bag (PRIMO VC9) / T2: purple & white, no bag (Bestron® D1400SP)*
PAW	30	O took dog's front paw (without command) and held it for 15 s, then repeated this for the other front paw (15 s)
UMBR	60	O walked towards T with the dog on leash (for security reasons), when dog was within ±1.5m T suddenly opened the umbrella and put it (opened) on the floor between T and O<br>*T1: black / T2: bright blue (both: ø 1 m)*
CYL	60	O sat next to T with the dog on leash (for security reasons), when the dog was looking away T suddenly shook the metal cylinder containing keys and put in on the floor between T and O<br>*T1: matte silver storage can (ø 7.5 x 16 cm) / T2: silver coffee pad can with colored prints (ø 7.5 x 18 cm)*
One last test was executed after all saliva samples had been taken		
FIST	60	Preparation (not incl. in analyses): O showed the dog a treat and closed his/her hand with the treat in it, once the dog touched the fist O opened his/her hand and gave the dog the treat<br>Test: same procedure, but now O held the fist closed for 60 s (afterwards O was allowed to give the treat in his/her own way, e.g., when the dog was calm or obeying an order)

T = Tester; O = Owner
Italic: different stimuli used at the puppy (T1) and young adult (T2) stage

2.3. Saliva sampling

Ten and forty minutes after the last stimulus presentation (metal cylinder) saliva samples were collected from the dogs. These time points were selected to represent dogs' physiological response to the test, as well as recovery, based on the reaction times of the selected salivary markers: Chromogranin A (CgA), cortisol and secretory immunoglobulin A (sIgA). Owners collected the samples to prevent influences of unfamiliar persons (Dreschel and Granger, 2005; Sandri et al., 2015). At each sampling occasion (pre-test, post10, post40), the owners put on clean latex gloves and collected saliva samples with four swabs, as described in the published article. The owners stored the samples in their freezer, and after the test these samples were transported on ice and stored within two hours (-20 °C). Sample processing and assaying are described in the published article.
In the periods between saliva sampling (i.e., test–post10, post10–post40) interaction with the dog was kept to a minimum. The tester questioned the owners about aspects that might have potentially biased the test results, and during this period owners could only interact with their dog if the dog actively sought attention. However, in the recovery period 10–40 minutes following the test, the dogs were allowed to move freely in the house and some dogs were allowed to urinate outside if needed. The intention of this period was to allow the dog to recover from the test, so activity (e.g., play, running) was avoided. Furthermore, the dogs could not yet eat or drink during this period, to prevent potential contamination of the final saliva sample (Salimetrics LLC and SalivaBio, 2013).

2.4. Ethics considerations

The described procedures (behavioral test, saliva sampling) were approved by the Ethical Commission of the University of Namur, who did not consider them animal experiments based on the non-invasive nature (as attested by seven international researchers, experienced with these types of measurement). The protocol was agreed upon by the Belgian State authority for Directive 2010/63/EU.
The owners were informed about the study aims and procedures, and signed a letter of informed consent. Their participation was voluntary, and they were told that they could interrupt the behavioral test at any moment. Also, the following endpoints were formulated to protect the welfare of the dogs:

Stop the subtest if:

- the dog panics (cowering, trembling, withdrawal, high vocalizations and/or non-responsive when the owner calls the dog), but recovers within 3 min (= interval between subtests: 1 min + 2 min pause);
- the dog shows mild threatening signals (fixed look, tonic immobility, growl-bark) towards a person

Stop the test if:

- the dog panics, but does not recover within 3 min
- the dog shows mild threatening signals towards a person for two consecutive tests

Exclude dog from the project if:

- the dog (attempts to) attack a person
- in case of panic during the test at the puppy stage, it was discussed with the owner whether or not the dog would be tested again at the young adult stage

References

De Meester, R.H., Pluijmakers, J., Vermeire, S., Laevens, H., 2011. The use of the socially acceptable behavior test in the study of temperament of dogs. Journal of Veterinary Behavior: Clinical Applications and Research 6, 211–224.

Dreschel, N.A., Granger, D.A., 2005. Physiological and behavioral reactivity to stress in thunderstorm-phobic dogs and their caregivers. Applied Animal Behaviour Science 95, 153–168.

Fratkin, J.L., Sinn, D.L., Patall, E.A., Gosling, S.D., 2013. Personality Consistency in Dogs: A Meta-Analysis. PLoS ONE 8, e54907.

Glenk, L.M., Kothgassner, O.D., Stetina, B.U., Palme, R., Kepplinger, B., Baran, H., 2014. Salivary cortisol and behavior in therapy dogs during animal-assisted interventions: A pilot study. Journal of Veterinary Behavior: Clinical Applications and Research 9, 98–106.

Hennessy, M.B., Williams, M.T., Miller, D.D., Douglas, C.W., Voith, V.L., 1998. Influence of male and female petters on plasma cortisol and behaviour: can human interaction reduce the stress of dogs in a public animal shelter? Applied Animal Behaviour Science 61, 63–77.

King, T., Hemsworth, P.H., Coleman, G.J., 2003. Fear of novel and startling stimuli in domestic dogs. Applied Animal Behaviour Science 82, 45–64.

Kobelt, A.J., Hemsworth, P.H., Barnett, J.L., Butler, K.L., 2003. Sources of sampling variation in saliva cortisol in dogs. Research in Veterinary Science 75, 157–161.

Lensen, C.M.M., Moons, C.P.H., Diederich, C., 2015. Saliva sampling in dogs: How to select the most appropriate procedure for your study. Journal of Veterinary Behavior: Clinical Applications and Research 10, 504-512.

Maros, K., Dóka, A., Miklósi, Á., 2008. Behavioural correlation of heart rate changes in family dogs. Applied Animal Behaviour Science 109, 329–341.

Palmer, R., Custance, D., 2008. A counterbalanced version of Ainsworth’s Strange Situation Procedure reveals secure-base effects in dog–human relationships. Applied Animal Behaviour Science 109, 306–319.

Pongrácz, P., Molnár, C., Miklósi, Á., Csányi, V., 2005. Human Listeners Are Able to Classify Dog (Canis familiaris) Barks Recorded in Different Situations. Journal of comparative psychology 119, 136–144.

Salimetrics L.L.C., SalivaBio, 2013. Saliva collection and handling advice, 3rd edition. https://www.salimetrics.com/assets/documents/Saliva_Collection _Handbook. (accessed 10/02/2014).

Sandri, M., Colussi, A., Perrotta, M.G., Stefanon, B., 2015. Salivary cortisol concentration in healthy dogs is affected by size, sex, and housing context. Journal of Veterinary Behavior: Clinical Applications and Research 10, 302–306.

Svartberg, K., Tapper, I., Temrin, H., Radesäter, T., Thorman, S., 2005. Consistency of personality traits in dogs. Animal Behaviour 69, 283–291.

Van den Berg, L., Schilder, M.B., de Vries, H., Leegwater, P.A., van Oost, B.A., 2006. Phenotyping of aggressive behavior in golden retriever dogs with a questionnaire. Behavior genetics 36, 882–902.

Van der Borg, J.A.M., Beerda, B., Ooms, M., de Souza, A.S., van Hagen, M., Kemp, B., 2010. Evaluation of behaviour testing for human directed aggression in dogs. Applied Animal Behaviour Science 128, 78–90.
