# Supplementary material for: Physiological stress reactivity and recovery related to behavioral traits in dogs (Canis familiaris)
Source: PLoS One. 2019 Sep 17;14(9):e0222581. doi: 10.1371/journal.pone.0222581 (PMC6748563; doi:10.1371/journal.pone.0222581)
Supplement: S1 Table — (PDF) [file pone.0222581.s003.pdf]

S1 Table

Overview of collected saliva volume (mean ± SD over three samples) and measured variables, for each dog at both test stages.

	T1 (puppy)				T2 (young adult)			
Breed	Volume (μL)	CgA	Cortisol	sIgA	Volume (μL)	CgA	Cortisol	sIgA
BSD	181 ± 252	Δ10	Δ40					
BSD	-							
X BSD / BMD	55 ± 52	Δ10 Δ40						
GSD	169 ± 156	Δ10 Δ40	Δ10 Δ40					
Dogo Argentino	68 ± 8	Δ10 Δ40	Δ10 Δ40	Δ10				
Great Dane	-	Δ10 Δ40		Δ10 Δ40				
Rottweiler	182 ± 74	Δ10 Δ40	Δ10 Δ40	Δ10 Δ40				
AmStaff	20 ± 25							
AmStaff	75 ± 3	Δ10 Δ40	Δ10 Δ40	Δ10 Δ40				
Engl. Cocker Sp.	104 ± 64	Δ10 Δ40		Δ40				
Labrador Retriever	40 ± 32*			Δ10				
Port. Water Dog	77 ± 29	Δ10 Δ40	Δ10 Δ40					
Shih Tzu	233 ± 189			Δ10 Δ40				
Shih Tzu	233 ± 21	Δ10 Δ40	Δ10 Δ40	Δ10 Δ40				
Whippet	15 ± 19*							
American Bulldog	129 ± 40		Δ10 Δ40					
Border Collie	80 ± 57*	Δ10 Δ40	Δ10 Δ40	Δ10 Δ40	461*	Δ10	Δ10	Δ10
Border Collie	88 ± 63		Δ10 Δ40	Δ10 Δ40	35 ± 23	Δ10 Δ40		Δ10 Δ40
Border Collie	-	Δ10 Δ40		Δ10 Δ40	43 ± 32	Δ10 Δ40	Δ10	Δ10 Δ40
Border Collie	236 ± 140	Δ10 Δ40	Δ10 Δ40	Δ10 Δ40	142 ± 32	Δ10 Δ40	Δ10 Δ40	
X B. Collie / Husky	130 ± 39	Δ10 Δ40	Δ10 Δ40	Δ10 Δ40	433 ± 146	Δ10 Δ40	Δ10 Δ40	Δ10 Δ40
BSD	-		Δ10 Δ40	Δ10 Δ40	136 ± 160			
BMD	75 ± 23	Δ40	Δ10 Δ40		41 ± 14		Δ40	
St. Bernard	98 ± 74		Δ10 Δ40		127 ± 48	Δ10 Δ40	Δ10 Δ40	Δ40
Dachshund	11 ± 9				30 ± 45			Δ10 Δ40
Engl. Cocker Sp.	31 ± 30	Δ10		Δ10	39 ± 41			
Am. Cocker Sp.	17 ± 18	Δ40			11 ± 15			
Fl. C. Retriever	95 ± 31	Δ10 Δ40	Δ10 Δ40		67 ± 25	Δ10 Δ40	Δ10 Δ40	
Boston Terrier	27 ± 38				29 ± 26	Δ10		
French Bulldog	-	Δ10 Δ40		Δ10	51 ± 35	Δ10 Δ40	Δ40	
X Tibetan Terrier	93 ± 25*	Δ10	Δ10		130 ± 97	Δ10	Δ10 Δ40	Δ10
X	110 ± 31	Δ10 Δ40	Δ10 Δ40	Δ10	133 ± 72	Δ40	Δ10 Δ40	Δ10 Δ40

-: mean volume could not be calculated due to missing data; * based on one (no SD) or two samples

Δ10 / Δ40: change in salivary stress marker concentration 10 / 40 min after the behavioral test compared to baseline (pre-test)
