# Supplementary material for: Physiological stress reactivity and recovery related to behavioral traits in dogs (Canis familiaris)
Source: PLoS One. 2019 Sep 17;14(9):e0222581. doi: 10.1371/journal.pone.0222581 (PMC6748563; doi:10.1371/journal.pone.0222581)
Supplement: S2 Table — (PDF) [file pone.0222581.s004.pdf]

S2 Table

Tested associations (Mann-Whitney U test) between dogs' physiological responses to a behavioral test (weak/strong) and C-BARQ scores (owner ratings).

Marker	Physiol. change	Marker range			C-BARQ	Weak response		Strong response		U	P	N	ES
		Group	Min	Max		(median ±	IQR)	(median ±	IQR)				
CgA	$\Delta10_{T1}$	Weak	-42.15	-0.49	SDA_{T1}	0.15	0.40	0.10	0.30	41.0	0.73	19	
		Strong	4.74	113.92	ODA_{T1}	0.13	0.51	0.13	0.25	41.5	0.77	19	
					DDA_{T1}	0.50	0.75	0.25	0.67	38.5	0.86	18	
					DDF_{T1}	0.88	1.00	0.75	0.33	40.5	0.71	19	
					RIV_{T1}	1.00	1.50	1.25	2.25	7.0	0.77	8	
					TRA_{T1}	2.50	0.88	2.50	0.25	39.0	0.62	19	
					CHA_{T1}	1.00	2.00	0.75	1.50	26.5	0.59	16	
					SDF_{T1}	0.25	0.25	0.00	0.50	42.0	0.79	19	
					NSF_{T1}	1.09	0.84	0.80	0.66	32.5	0.31	19	
					SEP_{T1}	1.07	0.39	0.63	0.25	17.5	**0.02**	19	0.83
					TCH_{T1}	0.71	1.25	0.50	0.75	26.0	0.38	17	
					EXC_{T1}	2.09	1.16	1.84	1.17	28.5	0.30	18	
					ATT_{T1}	2.09	0.83	1.83	1.00	41.0	0.74	19	
					NRG_{T1}	3.00	1.00	2.50	1.00	33.5	0.34	19	
CgA	$\Delta40_{T1}$	Weak	-60.67	-8.01	SDA_{T1}	0.15	0.25	0.05	0.30	39.0	0.89	18	
		Strong	2.91	61.85	ODA_{T1}	0.13	0.57	0.13	0.25	35.0	0.62	18	
					DDA_{T1}	0.00	0.88	0.25	0.67	33.5	0.51	18	
					DDF_{T1}	1.13	0.50	0.88	1.00	24.0	0.14	18	
					RIV_{T1}	1.50	1.50	0.50	1.00	4.5	0.37	8	
					TRA_{T1}	2.57	0.82	2.38	0.25	18.0	**0.04**	18	0.78
					CHA_{T1}	0.25	1.13	1.38	1.09	16.0	0.09	16	
					SDF_{T1}	0.25	0.50	0.13	0.50	39.5	0.93	18	
					NSF_{T1}	1.17	0.42	1.17	1.17	32.0	0.45	18	
					SEP_{T1}	0.94	0.50	0.76	0.50	39.5	0.93	18	
					TCH_{T1}	0.25	1.13	0.50	0.71	28.5	0.75	16	
					EXC_{T1}	2.09	1.17	1.00	1.33	19.5	0.11	17	
					ATT_{T1}	2.17	0.67	1.83	0.83	28.5	0.29	18	
					NRG_{T1}	2.50	1.25	2.75	1.50	39.0	0.89	18	
Cortisol	$\Delta10_{T1}$	Weak	-8.92	0.46	SDA_{T1}	0.00	0.35	0.24	0.25	20.0	0.19	16	
		Strong	0.50	4.43	ODA_{T1}	0.13	0.19	0.07	0.67	29.5	0.78	16	
					DDA_{T1}	0.25	0.75	0.59	0.63	30.0	0.55	17	
					DDF_{T1}	1.25	1.42	1.00	0.29	31.5	0.66	17	
					RIV_{T1}	0.00	0.25	0.50	1.00	5.5	0.22	9	
					TRA_{T1}	2.50	0.38	2.44	0.44	25.0	0.28	17	
					CHA_{T1}	0.33	2.25	1.25	1.59	27.5	0.95	15	
					SDF_{T1}	0.00	0.00	0.25	0.25	19.0	0.07	17	
					NSF_{T1}	1.09	1.09	1.09	0.94	28.0	0.67	16	
					SEP_{T1}	0.63	0.87	0.88	0.56	33.0	0.77	17	
					TCH_{T1}	0.63	0.84	1.00	1.50	25.0	0.72	15	
					EXC_{T1}	1.83	0.83	1.00	1.83	24.0	0.43	16	
					ATT_{T1}	2.17	0.67	1.59	0.59	16.0	0.05	17	
					NRG_{T1}	2.50	1.00	2.25	1.25	34.0	0.85	17	
Cortisol	$\Delta40_{T1}$	Weak	-7.45	1.94	SDA_{T1}	0.00	0.20	0.30	0.30	14.5	0.06	16	
		Strong	2.71	9.32	ODA_{T1}	0.13	0.13	0.00	0.63	31.0	0.95	16	
					DDA_{T1}	0.00	0.75	0.58	0.34	25.5	0.30	17	
					DDF_{T1}	0.50	1.25	1.00	0.13	30.0	0.56	17	
					RIV_{T1}	0.00	0.00	1.00	1.00	3.5	**0.04**	10	0.86

Marker	Physiol. change	Marker range			C-BARQ	Weak response		Strong response		U	P	N	ES
		Group	Min	Max		(median ±	IQR)	(median ±	IQR)				
					TRA$_{T1}$	2.50	0.38	2.44	0.50	28.5	0.46	17	
					CHA$_{T1}$	0.54	1.50	1.50	1.67	25.0	0.73	15	
					SDF$_{T1}$	0.00	0.00	0.25	0.25	14.5	**0.03**	17	0.80
					NSF$_{T1}$	1.00	0.50	1.17	1.00	29.0	0.79	16	
					SEP$_{T1}$	0.88	0.75	0.76	0.44	30.0	0.56	17	
					TCH$_{T1}$	0.50	1.00	0.63	1.50	24.5	0.76	15	
					EXC$_{T1}$	1.83	1.00	1.00	1.33	23.5	0.39	16	
					ATT$_{T1}$	2.17	0.67	1.50	1.16	18.0	0.08	17	
					NRG$_{T1}$	2.50	1.00	2.00	0.50	27.0	0.38	17	
sIgA	$\Delta 10_{T1}$	Weak	1.82	29.88	SDA$_{T1}$	0.15	0.30	0.00	0.30	22.0	0.53	15	
		Strong	-179.96	-18.23	ODA$_{T1}$	0.26	0.58	0.13	0.51	22.0	0.55	15	
					DDA$_{T1}$	0.67	0.50	0.25	0.50	17.0	0.23	15	
					DDF$_{T1}$	1.50	1.42	0.75	0.75	27.5	0.67	16	
					RIV$_{T1}$	0.50	4.00	0.00	1.50	5.5	0.53	8	
					TRA$_{T1}$	2.25	0.62	2.50	0.37	15.5	0.09	16	
					CHA$_{T1}$	1.25	1.67	0.25	1.13	16.0	0.55	13	
					SDF$_{T1}$	0.25	1.00	0.00	0.25	24.0	0.39	16	
					NSF$_{T1}$	1.09	0.67	1.17	0.84	24.0	0.72	15	
					SEP$_{T1}$	0.88	0.70	0.63	0.50	18.5	0.17	16	
					TCH$_{T1}$	0.75	0.25	0.33	0.50	11.0	0.12	14	
					EXC$_{T1}$	1.67	0.60	2.00	1.50	25.5	0.86	15	
					ATT$_{T1}$	2.17	0.83	2.00	0.67	26.5	0.59	16	
					NRG$_{T1}$	3.00	1.50	2.50	1.00	28.0	0.71	16	
sIgA	$\Delta 40_{T1}$	Weak	11.21	158.71	SDA$_{T1}$	0.00	0.00	0.15	0.20	6.5	0.10	11	
		Strong	-85.37	6.97	ODA$_{T1}$	0.13	0.25	0.44	0.57	9.0	0.27	11	
					DDA$_{T1}$	0.13	0.25	0.59	1.00	10.0	0.18	12	
					DDF$_{T1}$	0.46	1.25	1.38	1.33	7.5	0.09	12	
					RIV$_{T1}$	-	-	-	-	-	-	5	
					TRA$_{T1}$	2.32	0.13	2.57	0.37	8.0	0.10	12	
					CHA$_{T1}$	0.38	1.00	0.25	1.67	8.0	0.61	9	
					SDF$_{T1}$	0.00	0.00	0.25	0.25	11.0	0.21	12	
					NSF$_{T1}$	1.00	0.50	1.42	0.67	9.0	0.27	11	
					SEP$_{T1}$	0.63	0.38	1.07	0.87	13.5	0.47	12	
					TCH$_{T1}$	0.63	0.50	0.75	1.17	7.0	0.45	9	
					EXC$_{T1}$	1.42	1.00	2.00	0.83	9.5	0.31	11	
					ATT$_{T1}$	1.67	0.33	2.17	0.83	13.0	0.42	12	
					NRG$_{T1}$	2.25	1.50	2.50	1.00	18.0	1.00	12	
CgA	$\Delta 10_{T2}$	Weak	-58.14	-2.78	SDA$_{T2}$	0.75	0.80	0.45	0.44	7.5	0.33	10	
		Strong	7.72	47.98	ODA$_{T2}$	0.07	0.25	0.00	0.57	10.5	0.72	10	
					DDA$_{T2}$	0.67	1.00	1.00	1.00	10.5	0.75	10	
					DDF$_{T2}$	1.00	1.00	0.50	1.13	9.5	0.59	10	
					RIV$_{T2}$	-	-	-	-	-	-	3	
					TRA$_{T2}$	2.88	1.00	2.88	0.44	12.0	1.00	10	
					CHA$_{T2}$	2.42	2.17	1.75	1.42	9.5	0.59	10	
					SDF$_{T2}$	0.75	1.00	0.00	0.00	2.0	**0.02**	10	0.76
					NSF$_{T2}$	0.92	0.66	1.00	1.00	11.5	0.91	10	
					SEP$_{T2}$	0.94	0.37	0.44	1.01	8.5	0.45	10	
					TCH$_{T2}$	0.88	0.33	1.00	1.00	10.5	0.74	10	
					EXC$_{T2}$	2.00	0.83	1.59	1.17	7.0	0.28	10	

Marker	Physiol. change	Marker range			C-BARQ	Weak response		Strong response		U	P	N	ES
		Group	Min	Max		(median ±	IQR)	(median ±	IQR)				
					ATT_{T2}	2.92	1.16	2.00	1.09	4.5	0.11	10	
					NRG_{T2}	2.25	2.00	2.00	2.25	11.5	0.91	10	
CgA	$\Delta 40_{T2}$	Weak	-65.61	-4.43	SDA_{T2}	0.95	0.61	0.30	0.60	2.5	0.11	8	
		Strong	1.04	20.44	ODA_{T2}	0.13	0.25	0.00	0.13	6.0	0.49	8	
					DDA_{T2}	1.00	0.84	0.50	1.13	4.5	0.31	8	
					DDF_{T2}	0.75	1.00	0.63	1.75	7.0	0.76	8	
					RIV_{T2}	1.50	0.00	0.50	0.75	0.0	0.18	4	
					TRA_{T2}	2.94	0.44	2.75	1.07	6.0	0.56	8	
					CHA_{T2}	2.50	1.17	0.59	1.34	2.0	0.08	8	
					SDF_{T2}	0.25	1.50	0.50	1.13	8.0	1.00	8	
					NSF_{T2}	1.25	1.50	1.09	0.42	7.5	0.88	8	
					SEP_{T2}	1.13	0.38	0.44	0.94	2.0	0.08	8	
					TCH_{T2}	1.00	0.63	0.88	1.00	8.0	1.00	8	
					EXC_{T2}	2.58	0.92	1.42	0.59	0.0	**0.02**	8	1.00
					ATT_{T2}	3.00	0.97	2.34	0.75	3.5	0.19	8	
					NRG_{T2}	2.75	1.75	2.50	2.00	6.0	0.55	8	
Cortisol	$\Delta 10_{T2}$	Weak	-1.88	0.99	SDA_{T2}	0.50	0.80	0.84	0.56	7.0	0.77	8	
		Strong	1.36	3.35	ODA_{T2}	0.07	0.19	0.13	0.69	6.5	0.64	8	
					DDA_{T2}	0.13	0.75	1.13	0.71	2.5	0.11	8	
					DDF_{T2}	0.38	1.00	1.25	0.75	2.5	0.10	8	
					RIV_{T2}	0.75	1.50	0.50	0.00	1.0	0.65	4	
					TRA_{T2}	2.38	0.63	3.13	0.44	0.5	**0.03**	8	0.97
					CHA_{T2}	1.84	2.54	2.29	1.29	6.0	0.56	8	
					SDF_{T2}	0.13	0.75	0.50	1.75	7.0	0.76	8	
					NSF_{T2}	1.17	0.83	1.75	1.50	5.0	0.39	8	
					SEP_{T2}	0.32	1.01	1.07	0.26	4.0	0.25	8	
					TCH_{T2}	1.13	0.79	0.88	1.00	6.0	0.56	8	
					EXC_{T2}	2.09	1.25	1.92	1.42	7.5	0.88	8	
					ATT_{T2}	2.25	0.75	2.34	1.05	7.5	0.88	8	
					NRG_{T2}	2.00	2.50	3.25	1.25	3.0	0.14	8	
Cortisol	$\Delta 40_{T2}$	Weak	-2.22	0.32	SDA_{T2}	0.70	0.50	0.95	0.66	4.5	0.31	8	
		Strong	1.08	2.55	ODA_{T2}	0.07	0.19	0.13	0.25	7.0	0.75	8	
					DDA_{T2}	0.13	0.88	0.79	1.09	6.0	0.55	8	
					DDF_{T2}	0.25	0.38	1.25	0.63	3.5	0.18	8	
					RIV_{T2}	0.38	0.75	0.75	1.50	1.5	0.68	4	
					TRA_{T2}	2.44	0.69	2.88	0.50	2.5	0.11	8	
					CHA_{T2}	1.96	1.96	2.42	1.38	7.0	0.77	8	
					SDF_{T2}	0.88	1.38	0.50	1.75	6.0	0.56	8	
					NSF_{T2}	0.92	0.67	1.42	1.25	4.5	0.31	8	
					SEP_{T2}	0.82	0.69	1.07	0.88	5.5	0.47	8	
					TCH_{T2}	1.13	0.79	1.00	0.25	6.5	0.66	8	
					EXC_{T2}	2.08	0.67	2.00	2.00	8.0	1.00	8	
					ATT_{T2}	2.75	1.25	2.25	0.80	7.0	0.77	8	
					NRG_{T2}	1.50	1.75	3.00	0.50	0.0	**0.02**	8	1.00
sIgA	$\Delta 10_{T2}$	Weak	33.98	102.49	SDA_{T2}	0.69	0.89	0.50	1.00	4.5	0.59	7	
		Strong	-77.55	-4.87	ODA_{T2}	0.19	0.56	0.00	0.25	2.5	0.20	7	
					DDA_{T2}	0.75	1.25	1.00	0.50	5.5	0.86	7	
					DDF_{T2}	0.75	0.25	1.25	2.00	4.5	0.58	7	
					RIV_{T2}	-	-	-	-	-	-	2	

Marker	Physiol. change	Marker range			C-BARQ	Weak response		Strong response		U	P	N	ES
		Group	Min	Max		(median ±	IQR)	(median ±	IQR)				
					TRA_{T2}	2.63	0.38	3.13	0.38	0.0	**0.03**	7	1.00
					CHA_{T2}	2.63	1.50	1.25	2.50	3.0	0.29	7	
					SDF_{T2}	0.13	0.88	0.00	1.00	5.0	0.70	7	
					NSF_{T2}	1.67	1.17	0.67	0.67	2.5	0.21	7	
					SEP_{T2}	0.89	0.80	0.88	0.12	6.0	1.00	7	
					TCH_{T2}	1.38	0.79	0.25	0.75	1.0	0.08	7	
					EXC_{T2}	2.34	0.88	1.67	1.00	2.0	0.16	7	
					ATT_{T2}	2.17	0.42	2.50	0.50	2.0	0.15	7	
					NRG_{T2}	2.50	1.75	2.00	2.00	4.5	0.59	7	
sIgA	$\Delta 40_{T2}$	Weak	117.28	237.31	SDA_{T2}	0.20	1.60	0.50	1.00	4.5	1.00	6	
		Strong	-52.45	14.46	ODA_{T2}	0.13	0.25	0.00	0.25	3.5	0.64	6	
					DDA_{T2}	0.00	1.25	1.00	0.50	2.5	0.37	6	
					DDF_{T2}	0.75	1.25	1.25	2.00	2.5	0.38	6	
					RIV_{T2}	-	-	-	-	-	-	3	
					TRA_{T2}	2.50	1.00	3.13	0.38	0.0	**0.05**	6	1.00
					CHA_{T2}	1.00	2.75	1.25	2.50	4.0	0.83	6	
					SDF_{T2}	1.25	1.50	0.00	1.00	2.0	0.25	6	
					NSF_{T2}	1.50	0.50	0.67	0.67	0.0	**0.05**	6	1.00
					SEP_{T2}	0.29	1.38	0.88	0.12	3.0	0.51	6	
					TCH_{T2}	1.50	0.75	0.25	0.75	0.0	**0.05**	6	1.00
					EXC_{T2}	1.67	1.43	1.67	1.00	3.5	0.66	6	
					ATT_{T2}	2.33	1.00	2.50	0.50	4.0	0.82	6	
					NRG_{T2}	2.00	3.00	2.00	2.00	4.0	0.82	6	
CgA	$\Delta 10_{T1}$	Weak	-42.15	-0.49	SDA_{T2}	0.78	0.90	1.25	1.20	11.5	0.17	13	
		Strong	4.74	113.92	ODA_{T2}	0.25	0.88	0.07	0.38	17.0	0.55	13	
					DDA_{T2}	1.25	1.50	0.67	0.75	18.0	0.66	13	
					DDF_{T2}	1.00	1.00	1.00	0.75	21.0	1.00	13	
					RIV_{T2}	0.50	1.50	1.00	2.00	5.0	0.71	7	
					TRA_{T2}	2.75	0.50	2.19	0.26	10.5	0.13	13	
					CHA_{T2}	2.50	2.00	3.00	1.17	10.5	0.13	13	
					SDF_{T2}	0.00	0.50	1.75	2.25	4.0	**0.01**	13	0.91
					NSF_{T2}	1.00	1.16	1.17	1.00	20.5	0.94	13	
					SEP_{T2}	1.00	0.64	0.69	0.87	19.0	0.77	13	
					TCH_{T2}	1.00	1.00	0.84	0.75	18.0	0.66	13	
					EXC_{T2}	2.17	1.66	3.25	1.33	4.0	**0.02**	13	0.91
					ATT_{T2}	2.17	0.67	2.92	1.43	12.0	0.20	13	
					NRG_{T2}	3.00	0.50	2.75	1.50	21.0	1.00	13	
CgA	$\Delta 40_{T1}$	Weak	-60.67	-8.01	SDA_{T2}	0.80	0.80	0.89	1.00	11.0	0.29	12	
		Strong	2.91	61.85	ODA_{T2}	0.00	0.25	0.25	1.13	11.5	0.29	12	
					DDA_{T2}	1.00	1.25	1.00	1.00	17.0	0.93	12	
					DDF_{T2}	1.25	2.00	0.75	1.25	11.5	0.32	12	
					RIV_{T2}	-	-	-	-	-	-	7	
					TRA_{T2}	2.88	0.25	2.50	0.63	7.0	0.09	12	
					CHA_{T2}	2.50	1.42	2.33	1.00	14.5	0.62	12	
					SDF_{T2}	1.00	1.25	0.50	2.50	15.5	0.74	12	
					NSF_{T2}	1.17	0.50	1.67	1.33	7.0	0.08	12	
					SEP_{T2}	1.00	0.12	1.14	1.00	13.0	0.46	12	
					TCH_{T2}	0.75	0.33	1.00	1.25	12.5	0.41	12	
					EXC_{T2}	1.67	0.50	2.83	1.16	5.0	**0.04**	12	0.71

Marker	Physiol. change	Marker range			C-BARQ	Weak response		Strong response		U	P	N	ES
		Group	Min	Max		(median ±	IQR)	(median ±	IQR)				
					ATT_{T2}	2.50	1.00	2.33	1.43	16.5	0.87	12	
					NRG_{T2}	2.00	0.50	3.00	1.00	4.5	**0.03**	12	0.71
Cortisol	$\Delta 10_{T1}$	Weak	-8.92	0.46	SDA_{T2}	0.75	0.50	0.69	0.20	17.5	0.94	12	
		Strong	0.50	4.43	ODA_{T2}	0.09	0.25	0.07	0.38	16.5	0.80	12	
					DDA_{T2}	1.00	0.92	0.25	1.00	11.0	0.26	12	
					DDF_{T2}	0.75	1.25	0.63	0.50	17.0	0.87	12	
					RIV_{T2}	-	-	-	-	-	-	6	
					TRA_{T2}	2.94	0.38	2.44	0.38	6.5	0.07	12	
					CHA_{T2}	1.79	1.50	2.25	1.75	16.5	0.81	12	
					SDF_{T2}	0.50	1.25	0.38	2.25	16.0	0.74	12	
					NSF_{T2}	1.00	1.33	1.09	0.66	16.0	0.75	12	
					SEP_{T2}	1.01	0.37	0.88	0.51	15.0	0.63	12	
					TCH_{T2}	0.88	0.92	0.84	1.00	17.5	0.94	12	
					EXC_{T2}	1.84	1.16	2.25	0.67	15.0	0.63	12	
					ATT_{T2}	2.67	1.00	2.17	0.17	5.0	**0.04**	12	0.85
					NRG_{T2}	3.00	2.00	2.75	1.00	17.5	0.93	12	
Cortisol	$\Delta 40_{T1}$	Weak	-7.45	1.94	SDA_{T2}	0.70	0.60	0.79	1.00	12.5	0.38	12	
		Strong	2.71	9.32	ODA_{T2}	0.00	0.25	0.09	0.38	14.0	0.47	12	
					DDA_{T2}	0.54	1.25	1.25	1.75	11.0	0.26	12	
					DDF_{T2}	0.75	1.25	0.63	1.00	16.0	0.74	12	
					RIV_{T2}	0.75	1.50	0.00	1.00	4.5	0.56	7	
					TRA_{T2}	2.88	0.63	2.51	0.62	10.5	0.23	12	
					CHA_{T2}	1.79	1.83	2.25	1.75	14.5	0.57	12	
					SDF_{T2}	0.50	1.25	1.38	2.75	13.0	0.41	12	
					NSF_{T2}	1.17	1.16	1.09	0.66	17.0	0.87	12	
					SEP_{T2}	0.88	1.25	1.07	0.39	14.0	0.52	12	
					TCH_{T2}	1.00	0.50	0.67	1.00	16.0	0.75	12	
					EXC_{T2}	1.84	1.66	2.25	1.34	11.5	0.30	12	
					ATT_{T2}	2.50	1.16	2.42	0.50	12.0	0.33	12	
					NRG_{T2}	3.00	2.00	3.00	1.00	14.5	0.56	12	
sIgA	$\Delta 10_{T1}$	Weak	1.82	29.88	SDA_{T2}	0.60	1.00	0.84	0.50	13.0	0.71	11	
		Strong	-179.96	-18.23	ODA_{T2}	0.38	0.63	0.13	0.25	7.0	0.14	11	
					DDA_{T2}	1.00	1.00	1.13	0.75	12.0	0.58	11	
					DDF_{T2}	0.50	1.00	1.00	1.00	9.0	0.27	11	
					RIV_{T2}	-	-	-	-	-	-	4	
					TRA_{T2}	2.38	1.00	3.07	0.50	3.0	**0.03**	11	0.83
					CHA_{T2}	2.50	1.00	2.29	1.25	10.0	0.36	11	
					SDF_{T2}	0.00	1.00	0.25	1.00	15.0	1.00	11	
					NSF_{T2}	0.83	1.00	0.92	1.66	13.0	0.71	11	
					SEP_{T2}	1.14	0.88	1.00	0.26	13.5	0.78	11	
					TCH_{T2}	0.33	1.00	0.88	0.75	15.0	1.00	11	
					EXC_{T2}	2.83	0.34	2.09	0.66	7.5	0.17	11	
					ATT_{T2}	2.67	0.84	2.59	1.16	14.0	0.85	11	
					NRG_{T2}	3.00	0.50	2.75	1.50	14.5	0.92	11	
sIgA	$\Delta 40_{T1}$	Weak	11.21	158.71	SDA_{T2}	0.60	1.30	0.78	0.29	7.5	1.00	8	
		Strong	-85.37	6.97	ODA_{T2}	0.17	1.63	0.25	0.38	7.0	0.88	8	
					DDA_{T2}	1.00	1.25	1.00	0.92	5.5	0.55	8	
					DDF_{T2}	0.00	0.25	1.25	0.50	0.0	**0.02**	8	1.00
					RIV_{T2}	-	-	-	-	-	-	3	

Marker	Physiol. change	Marker range			C-BARQ	Weak response (median ± IQR)		Strong response (median ± IQR)		U	P	N	ES
		Group	Min	Max									
					TRA_{T2}	2.88	1.38	3.00	0.50	5.0	0.46	8	
					CHA_{T2}	1.25	2.50	2.25	0.08	7.0	0.88	8	
					SDF_{T2}	0.00	1.00	1.00	2.50	4.5	0.34	8	
					NSF_{T2}	0.50	1.50	1.17	1.66	3.0	0.18	8	
					SEP_{T2}	0.88	0.76	1.14	0.25	3.0	0.17	8	
					TCH_{T2}	0.33	1.50	0.75	0.75	6.0	0.65	8	
					EXC_{T2}	2.00	2.33	2.17	1.50	6.5	0.76	8	
					ATT_{T2}	2.67	1.33	2.50	1.00	4.0	0.29	8	
					NRG_{T2}	2.50	2.00	3.00	1.00	4.0	0.29	8	

The upper panel presents data from T1 (puppy stage), the middle panel from T2 (young adult stage), the lower panel combines physiological data from T1 with C-BARQ data from T2 (prediction). Dogs with a “strong response” have the largest physiological change (above median), as expected for that marker (increase for CgA and cortisol, decrease for sIgA).

Δ10 / Δ40: change in salivary marker concentration 10/40 min after the behavioral test, compared to pre-test

Marker range: min – max change in salivary marker concentration for each marker, at the specified sample moment (Δ10 / Δ40) and per group; measurement units: pmol/mL (CgA), nmol/L (cortisol), EU/mL (sIgA; EU = Elisa Units).

SDA: Stranger-directed aggression; ODA: Owner-directed aggression; DDA: Dog-directed aggression; DDF: Dog-directed fear; RIV: Dog rivalry; TRA: Trainability; CHA: Chasing; SDF: Stranger-directed fear; NSF: Non-social fear; SEP: Separation-related behavior; TCH: Touch sensitivity; EXC: Excitability; ATT: Attachment and attention-seeking; NRG: Energy. C-BARQ scores derived from questionnaire scales (0–4, no measurement unit).

ES: effect size (probabilistic index), calculated for significant results only

- : analyses could not be performed with maximum two dogs in one group (weak/strong response)

bold: P-values < 0.05
